# Non-Small Cell Lung Cancer Survival by Race and Ethnicity in California, 2014-2019 Differences by Sex and Smoking History

**DOI:** 10.1016/j.chpulm.2025.100190

**Published:** 2025-06-20

**Authors:** Jeffrey B. Velotta, Julie Von Behren, Rachel A. Allison, Linda Shin, Iona Cheng, Scarlett Lin Gomez

**Affiliations:** a Division of Thoracic Surgery, Kaiser Permanente Oakland Medical Center, Kaiser Permanente Northern California, Oakland, CA; b Department of Surgery, School of Medicine, University of California San Francisco, San Francisco, CA; c Department of Epidemiology and Biostatistics, University of California San Francisco, San Francisco, CA; d Helen Diller Family Comprehensive Cancer Center, University of California San Francisco, San Francisco, CA; e Greater Bay Area Cancer Registry, University of California San Francisco, San Francisco, CA; f Department of Clinical Science, Kaiser Permanente Bernard J. Tyson School of Medicine, Pasadena, CA; g UCSF School of Medicine, San Francisco, CA

**Keywords:** Asian American, lung cancer, mortality

## Abstract

**Background:**

Lung cancer is currently the leading cause of cancer-related death. There are gaps in knowledge regarding survival patterns according to sex and smoking status across racial and ethnic groups.

**Research Question:**

Are there mortality differences according to race/ethnicity among individuals diagnosed with lung cancer in California when stratified by sex and smoking status?

**Study Design and Methods:**

Non-small cell lung cancer (NSCLC) cases diagnosed from 2014 through 2019 from the California Cancer Registry, a population-based cancer surveillance system, were analyzed. Using Cox regression models, hazard ratios (HRs) were estimated for NSCLC mortality according to race and ethnicity, stratified by sex and smoking status and adjusted for sociodemographic and clinical variables.

**Results:**

Among 28,854 female individuals, every racial and ethnic group had an HR < 1 compared with non-Hispanic White (NHW) individuals, with the exception of Native Hawaiian and Pacific Islander and American Indian and Alaskan Native (AIAN) populations, who had comparable mortality. Non-Hispanic Black, Hispanic, and Chinese female individuals had lower mortality relative to NHW female individuals, with HRs of 0.94 (95% CI, 0.89-0.99), 0.89 (95% CI, 0.85-0.93), and 0.82 (95% CI, 0.76-0.88), respectively. Among 29,499 male individuals, every racial and ethnic group exhibited a lower HR, compared with NHW male individuals, with the exception of Japanese (HR, 1.14; 95% CI, 0.99-1.31) and AIAN (HR, 1.15; 95% CI, 0.96-1.38) male individuals. Vietnamese female individuals who had never smoked had lower mortality (HR, 0.80; 95% CI, 0.69-0.92) relative to NHW female individuals. Chinese male individuals who had never smoked had lower mortality (HR, 0.83; 95% CI, 0.71-0.97) compared with NHW male individuals who had never smoked.

**Interpretation:**

In this population-based study of NSCLC, we found mortality differences across racial/ethnic groups when accounting for sex and smoking status. Further research focused on mortality differences in NSCLC stratified according to race and ethnicity should focus on social determinants of health and health care access factors.

**Take-Home Points**

**Study Question:** Are there mortality differences by race/ethnicity among individuals diagnosed with lung cancer in California when stratified according to sex and smoking status?

**Results:** Among 28,854 female individuals, every racial and ethnic group had an HR < 1 compared with non-Hispanic White individuals, with the exception of Native Hawaiian and Pacific Islander and American Indian and Alaskan Native individuals, who had comparable mortality.

**Interpretation:** In this population-based study of non-small cell lung cancer, we found mortality differences across racial/ethnic groups when accounting for sex and smoking status.

Lung cancer is currently the leading cause of cancer-related death in the United States,^1^ with a 5-year relative survival rate of 26.7%.^2^ Race and ethnicity have been considered to be associated with lung cancer survival because of disparities that exist in the United States regarding cancer screening and treatment.^3^, ^4^, ^5^ Of note, Black individuals with lung cancer are 15% less likely to be diagnosed early, 11% more likely to receive no treatment, and 16% less likely to survive 5 years, compared with non-Hispanic White (NHW) individuals.^3^ In past decades, Black male individuals in the United States had significantly higher population-level lung cancer mortality rates compared with NHW male individuals, although, notably, these differences have lessened considerably such that they are nearly comparable in the latest years of available data.^6^ In the latest Annual Report to the Nation, population-level mortality rates of lung cancer for 2018 to 2022 were 46.7 and 41.2 per 100,000 in Black and White male individuals, respectively; however, incidence rates (2017-2021) were 71.5 and 64.6 per 100,000, respectively, suggesting that much of the mortality differentials are due to incidence rather than differential death rates following diagnosis.

In addition, Surveillance, Epidemiology, and End Results (SEER) data have shown improved mortality rates in American Indian, Asian American, and Hispanic patients compared with NHW patients with lung cancer.^6^ Studies of lung cancer mortality following diagnosis have also shown similar or lower mortality among Asian American, Black, and Hispanic individuals compared with NHW individuals once clinical and demographic factors were taken into account.^7^, ^8^, ^9^ Most studies that have assessed lung cancer outcomes have presented data for Asian American, Native Hawaiian, and Pacific Islander (AANHPI) populations as an aggregated group, despite the fact that AANHPI represents populations from > 40 countries. In addition, AANHPI individuals are highly heterogeneous regarding ancestry, sociodemographic differences, immigration history, and cancer risk factor profiles.^10^, ^11^, ^12^ California has the largest number of AANHPI individuals of any state in the United States, providing an opportunity to examine disaggregated data to better understand and address how distinct AANHPI ethnic groups experience cancer disparities.^13^, ^14^, ^15^

A patient’s smoking status affects lung cancer mortality, with patients who smoke or have smoked in the past having higher mortality than those who have never smoked.^16^ Due to prior reported differences in overall survival between patients who have never smoked and people who have ever smoked in NSCLC, it is important to take into account smoking status in overall mortality analyses as it pertains to different races and/or ethnicities. Moreover, there is reported variability but increasing prevalence of never smoking-associated lung cancer among AANHPI individuals, especially female individuals; there is concern that a distinctly unique profile of lung cancer diagnoses may exist in these populations.^17^ In particular, the majority of Asian American women with lung cancer have never smoked, with reported heterogeneity across ethnic groups, with a rate of nearly 80% among Chinese and American women of Asian Indian descent. In addition, the Asian American population who had ever smoked also exhibits extreme heterogeneity as shown in a recent study from the Jefferson Lung Cancer Screening (LCS) Program Registry, which found significantly lower LCS adherence rates in Asian American individuals compared with other racial and ethnic groups.^18^

Given the recent availability of smoking information in the California Cancer Registry, along with a large racially and ethnically diverse population, we have the unique opportunity to examine mortality patterns across racial/ethnic groups, with attention to detailed AANHPI ethnicities, and the extent of racial and ethnic differences by smoking status and sex.

## Study Design and Methods

### Demographic and Clinical Information

Sociodemographic and clinical characteristics were obtained from the California Cancer Registry. This registry has detailed demographic and clinical information on all invasive cancers diagnosed in the state since 1988.^19^ These factors included age at diagnosis, race and ethnicity, sex, tobacco smoking history (never, former, current), year of diagnosis, American Joint Committee on Cancer (AJCC) stage, Charlson Comorbidity Index scores (based on linkage to statewide hospitalization data from the California Department of Health Care Access and Information), surgery treatment (yes, no), chemotherapy treatment (yes, no), radiation treatment (yes, no), insurance type (no insurance, private only, Medicare only or Medicare + private, any Medicaid, any military/other public, unknown) determined from primary and secondary payer sources, whether the patient was seen at a National Cancer Institute (NCI)-designated cancer center (yes, no), and marital status (never married, married or domestic partner, separated/divorced/widowed, unknown). Race/ethnicity was categorized into 13 mutually exclusive groups: American Indian and Alaska Native (AIAN), Chinese, Filipino, Hispanic, Japanese, Korean, Native Hawaiian and Pacific Islander (NHPI), non-Hispanic Black (NHB), NHW (reference group), Other Asian, South Asian, Southeast Asian (excluding Vietnamese), and Vietnamese.

Address at diagnosis was geocoded and linked at a census tract level to a widely used index of neighborhood socioeconomic status.^20^, ^21^, ^22^, ^23^ The neighborhood socioeconomic status composite index was created from principal component analysis of 7 indicators from the US Census American Community Survey (2007-2011): median household income, Liu education index (among individuals aged ≥ 25 years), percentage below 200% of poverty line, proportion with blue collar occupation, proportion without a job, median rent, and median house value.^24^

### NSCLC Cases

The NSCLC cases diagnosed from 2014 to 2019 (N = 92,280) were selected from the California Cancer Registry database (based on data available as of January 2024). Based on International Classification of Diseases for Oncology, Third Edition, codes, cases classified as adenocarcinoma, squamous cell, large cell, other specified carcinoma, and unspecified carcinoma were included. Cases that were not primary cancers (n = 23,358) and were not invasive (n = 245) were excluded. The outcomes examined were deaths from any cause. Follow-up information on vital status of the cases and the cause of death was obtained through routine linkages to state and national mortality files. The time to event (follow-up time) was calculated in days from the date of cancer diagnosis to the time of death, last known follow-up, or end of study follow-up period (December 31, 2021), whichever came first.

This study was approved for humans under the Greater Bay Area Cancer Registry Institutional Review Board protocol at the University of California San Francisco.

### Statistical Analysis

Hazard ratios (HRs) and 95% CIs were estimated by using Cox proportional hazards regression models with clustering by census tract to account for correlation among participants living in the same tracts. All of the demographic and clinical factors included in the multivariable models were originally selected a priori and were statistically significant in univariate associations with the outcomes of interest (based on χ^2^ tests). The proportional hazards assumption was tested using the correlation between time and scaled Schoenfeld residuals for each covariate. Because the proportional hazards assumption was violated for stage, receipt of radiation, and receipt of chemotherapy, we used the strata function in the models for stage, radiation (as yes, no, or unknown), and chemotherapy (as yes, no, or unknown); this function in SAS allows the hazards to vary across stratum or levels. Multiple imputation was used to impute smoking information for the 31% of cases with unknown tobacco smoking history. Maximum likelihood logistic regression and imputed smoking information was used for male and female individuals separately using the demographic and clinical factors described earlier. The imputation models based on the Rubin strategy were fitted 20 times and the parameter estimates averaged across the imputations.^25^

All analyses were performed in SAS version 9.4 (SAS Institute, Inc).

## Results

A total of 58,353 incident primary NSCLC cases and 41,905 deaths from any cause were included in this analysis. The cases were equally divided between female and male groups (49.4% vs 50.6%, respectively). The number of cases and deaths according to sociodemographic and clinical factors for female individuals are shown in Table 1. Most of the female individuals were aged > 60 years at diagnosis (83%). They were ethnically diverse, with 63% NHW, 13% Hispanic, 8% Black, 5% Chinese, 4% Filipino, 2% Vietnamese, 1% Japanese, 1% Korean, and < 1% for the remaining groups, which included Southeast Asian, NHPI, and AIAN. Stage IV was the most common stage of diagnosis (51%); the majority of NSCLC cases were classified as adenocarcinomas (65%), with squamous cell carcinoma being the second most common type (16%). Overall, 20% of female patients had never smoked, 49% of patients currently smoked or formerly smoked, and 32% of patients were missing information on smoking history. Using imputed values for smoking history, 24% of female patients were classified as having never smoked and 76% were classified as having currently or formerly smoked.

**Table 1:** NSCLC Cases, Demographic and Clinical Characteristics: Female Individuals Diagnosed 2014 to 2019 in California

Characteristic	Cases	% Overall	Deaths Due to Any Cause^a^	% Mortality
Total	28,854	100	19,394	67
Age category at diagnosis, y				
< 50	1,163	4.0	583	50
50-59	3,793	13.1	2,333	62
60-69	8,276	28.7	5,137	62
70-79	9,799	34.0	6,689	68
≥ 80	5,823	20.2	4,652	80
Race/ethnicity				
Non-Hispanic White	18,118	62.8	12,435	69
Non-Hispanic Black	2,315	8.0	1,679	73
Hispanic	3,874	13.4	2,550	66
Chinese	1,441	5.0	820	57
Japanese	285	1.0	197	69
Filipino	1031	3.6	587	57
Korean	279	1.0	174	62
Vietnamese	624	2.2	379	61
Southeast Asian^b^	165	0.6	110	67
South Asian^c^	160	0.6	81	51
Other Asian^d^	189	0.7	111	59
Native Hawaiian, Pacific Islander	152	0.5	104	68
American Indian/Alaska Native	221	0.8	167	76
AJCC stage				
I	7,269	25.2	2,286	31
II	2,130	7.4	1,117	52
III	4,841	16.8	3,331	69
IV	14,614	50.6	12,660	87
Histologic type (ICD-O-3)				
Unspecified carcinoma	3,282	11.4	2,848	87
Large cell + other specified carcinoma	2,411	8.4	1,267	53
Adenocarcinoma	18,655	64.7	11,844	63
Squamous cell	4,506	15.6	3,435	76
Surgery				
No	21,208	73.5	17,272	81
Yes	7,631	26.4	2,114	28
Unknown	15	0.1	8	53
Chemotherapy				
No	17,204	59.6	10,995	64
Yes	11,202	38.8	8,064	72
Unknown	448	1.6	335	75
Radiation treatment				
No	19,232	66.7	12,332	64
Yes	9,605	33.3	7,051	73
Unknown	17	0.1	11	65
Charlson Comorbidity Index score				
0	7,836	27.2	4,785	61
1	7,684	26.6	5,096	66
≥ 2	8,028	27.8	6,188	77
Unknown	5,306	18.4	3,325	63
Tobacco smoking history				
Unknown	9,121	31.6	6,065	66
Never	5,732	19.9	3,393	59
Current or former	14,001	48.5	9,936	71
Imputed smoking history				
Never	6,978	24.2	3,866	55
Current or former	21,876	75.8	15,528	71
Health insurance status^e^				
No insurance	251	0.9	198	79
Private only	9,663	33.5	5,999	62
Medicare only or Medicare + private	12,712	44.1	8,691	68
Any Medicaid	4,990	17.3	3,617	72
Any military/other public	721	2.5	491	68
Unknown	517	1.8	398	77
NCI-designated cancer center				
Not NCI	24,882	86.2	17,222	69
Seen at NCI center	3,972	13.8	2,172	55
Marital status at diagnosis				
Never married	4,964	17.2	3,403	69
Married or domestic partner	12,125	42.0	7,529	62
Separated/divorced/widow	10,832	37.5	7,823	72
Unknown	933	3.2	639	68
Neighborhood SES quintile				
Unknown	880	3.0	578	66
Quintile 1 (lowest)	4,162	14.4	3,055	73
Quintile 2	5,743	19.9	4,021	70
Quintile 3	6,167	21.4	4,231	69
Quintile 4	6,206	21.5	4,027	65
Quintile 5 (highest)	5,696	19.7	3,482	61

Data are presented as No. or %. AJCC = American Joint Committee on Cancer; ICD-O-3 = International Classification of Diseases for Oncology, Third Edition; NCI = National Cancer Institute; NSCLC = non-small cell lung cancer; SES = socioeconomic status based on census tract of residence.

a Based on last known follow-up time through December 31, 2021.

b Lao, Hmong, Kampuchean, Cambodian, and Thai.

c Indian, Pakistani, Sri Lankan, and Bangladeshi.

d Bhutanese, Nepalese, Sikkimese, Burmese, Indonesian, and other Asian, Asian not otherwise specified.

e Health insurance coverage based on primary and secondary payer source.

The number of cases and deaths according to sociodemographic and clinical factors for male individuals is presented in Table 2. Similar to female patients, male patients were mostly aged > 60 years at diagnosis (83%). They were classified as 60% NHW, 14% Hispanic, 8% Non-Hispanic black, 5% Chinese, 4% Filipino, 3% Vietnamese, 1% Korean, and < 1% for the remaining groups, Japanese, Southeast Asian, NHPI, and AIAN. More than one-half of the cases were stage IV (54%), and the most common histologic type was adenocarcinoma (54%).

**Table 2:** NSCLC Cases, Demographic and Clinical Characteristics: Male Individuals Diagnosed 2014 to 2019 in California

Characteristic	Cases	% Overall	Deaths Due to Any Cause^a^	% Mortality
All	29,499	100.0	22,511	76
Age category at diagnosis, y				
< 50	989	3.4	588	59
50-59	3,901	13.2	2,823	72
60-69	9,511	32.2	6,936	73
70-79	9,904	33.6	7,669	77
≥ 80	5,194	17.6	4,495	87
Race/ethnicity				
Non-Hispanic White	17,802	60.3	13,680	77
Non-Hispanic Black	2,333	7.9	1,873	80
Hispanic	4,034	13.7	3,160	78
Chinese	1,557	5.3	1,033	66
Japanese	245	0.8	200	82
Filipino	1,219	4.1	896	74
Korean	399	1.4	293	73
Vietnamese	950	3.2	658	69
Southeast Asian^b^	176	0.6	142	81
South Asian^c^	261	0.9	180	69
Other Asian^d^	168	0.6	118	70
Native Hawaiian, Pacific Islander	158	0.5	119	75
American Indian/Alaska Native	197	0.7	159	81
AJCC stage				
I	5,621	19.1	2,412	43
II	2,225	7.5	1,340	60
III	5,611	19.0	4,249	76
IV	16,042	54.4	14,510	90
Histologic type (ICD-O-3)				
Unspecified carcinoma	3,946	13.4	3,506	89
Large cell + other specified carcinoma	2,327	7.9	1,616	69
Adenocarcinoma	15,954	54.1	11,671	73
Squamous cell	7,272	24.7	5,718	79
Surgery				
No	23,277	78.9	20,041	86
Yes	6,199	21.0	2,453	40
Unknown	23	0.1	17	74
Chemotherapy				
No	16,953	57.5	12,758	75
Yes	11,991	40.6	9,298	78
Unknown	555	1.9	455	82
Radiation treatment				
No	19,086	64.7	14,283	75
Yes	10,393	35.2	8,211	79
Unknown	20	0.1	17	85
Charlson Comorbidity Index score				
0	6,096	20.7	4,358	71
1	7,400	25.1	5,590	76
≥ 2	10,447	35.4	8,700	83
Unknown	5,556	18.8	3,863	70
Tobacco smoking history				
Unknown	9,560	32.4	7,303	76
Never	2,632	8.9	1,789	68
Current or former	17,307	58.7	13,419	78
Imputed smoking history				
Never	2,679	9.1	1,800	67
Current or former	26,820	90.9	20,711	77
Health insurance status^e^				
No insurance	332	1.1	268	81
Private only	9,102	30.9	6,458	71
Medicare only or Medicare + private	11,692	39.6	9,126	78
Any Medicaid	6,115	20.7	4,906	80
Any military/other public	1,679	5.7	1,270	76
Unknown	579	2.0	483	83
NCI-designated cancer center				
Not NCI	25,149	85.3	19,546	78
Seen at NCI center	4,350	14.7	2,965	68
Marital status at diagnosis				
Never married	5,551	18.8	4,491	81
Married or domestic partner	17,629	59.8	12,908	73
Separated/divorced/widow	5,380	18.2	4,373	81
Unknown	939	3.2	739	79
Neighborhood SES quintile				
Unknown	1,100	3.7	807	73
Quintile 1 (lowest)	4,826	16.4	3,980	82
Quintile 2	5,973	20.2	4,720	79
Quintile 3	6,405	21.7	4,934	77
Quintile 4	6,078	20.6	4,567	75
Quintile 5 (highest)	5,117	17.3	3,503	68

Data are presented as No. or %. AJCC = American Joint Committee on Cancer; ICD-O-3 = International Classification of Diseases for Oncology, Third Edition; NCI = National Cancer Institute; NSCLC = non-small cell lung cancer; SES = socioeconomic status based on census tract of residence.

a Based on last known follow-up time through December 31, 2021.

b Lao, Hmong, Kampuchean, Cambodian, and Thai.

c Indian, Pakistani, Sri Lankan, and Bangladeshi.

d Bhutanese, Nepalese, Sikkimese, Burmese, Indonesian, and other Asian, Asian not otherwise specified.

e Health insurance coverage based on primary and secondary payer source.

No substantial differences were observed in the percentage of lung cancer cases with unknown smoking information when characteristics such as sex age, race, and stage at diagnosis were examined (e-Table 1), with the most notable differences seen across racial/ethnic groups. The percentage of cases with unknown smoking information ranged from 26% unknown among AIAN to 40% among Filipino and South Asian groups. Cases with missing smoking status data were generally similar to cases with available information, when considering the approximate average of the distributions of characteristics between individuals who never smoked and those who ever smoked. The only difference noted was a slightly lower percentage of individuals (11.3%) seen at an NCI cancer center among those with unknown smoking history data, relative to those who had never and those who had ever smoked (21.1% and 14.2% seen at an NCI cancer center, respectively). The similar characteristics between patients with known and unknown smoking information suggests that smoking status is mostly missing at random, and thus our multiple imputation approach is reasonable.

**Figure 1:**
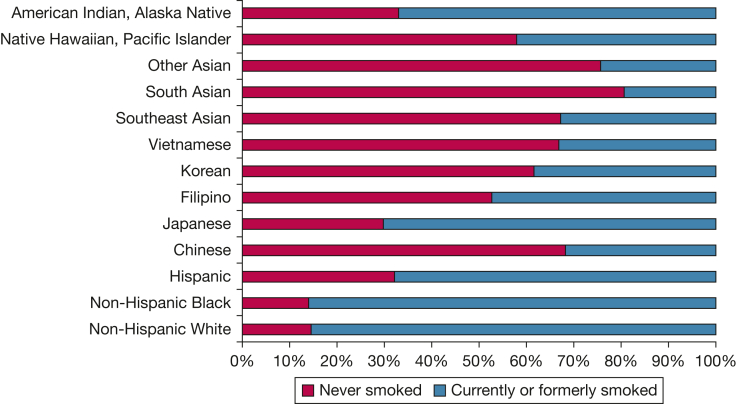
Smoking status (imputed for missing) according to race and ethnicity in female individuals diagnosed with non-small cell lung cancer from 2014 to 2019 in California.

**Figure 2:**
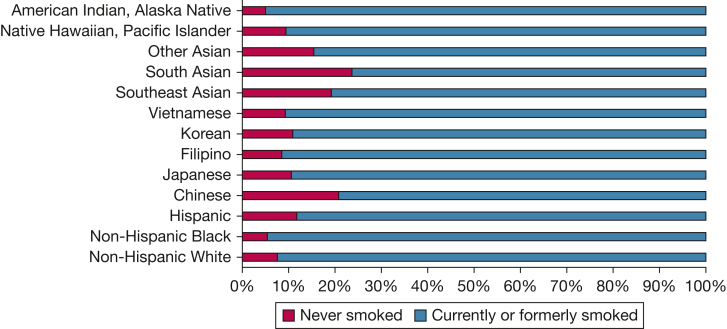
Smoking status (imputed for missing) according to race and ethnicity in male individuals diagnosed with non-small cell lung cancer from 2014 to 2019 in California.

When we examined smoking history patterns using imputed values for missing smoking history, pronounced differences were observed according to both sex and race/ethnicity (Figure 1, Figure 2). The proportion of those who had never smoked was higher among female patients (24%) compared with male patients (9%). Among female patients, the highest prevalence of never smoking was observed among South Asian (81%), other Asian (76%), Chinese (68%), and Southeast Asian (67%) individuals. Among male patients, the highest prevalence of never smoking was seen among South Asian (24%), Chinese (21%), and Southeast Asian (19%) populations. The highest proportion of female patients who currently smoke or smoked in the past was among NHW (86%), NHB (85%), and Japanese (70%) populations. The highest proportion of those who currently smoke or smoked in the past for male patients was in AIAN (95%), NHB (95%), and NHW (93%) individuals.

Kaplan-Meier curves for survival probabilities across racial and ethnic groups by sex, and additionally by sex and smoking status, are shown in e-Figures 1 to 6. Among female patients overall, South Asian individuals had the highest while AIAN groups had the lowest percentage surviving at 3 years (56.9% and 30.3%, respectively). Among male patients overall, Chinese individuals had the highest percentage surviving at 3 years (39.4%), whereas Japanese and AIAN groups had the lowest (22.0% and 21.7%). These patterns were generally similar according to smoking status.

The adjusted HRs for all-cause mortality among female patients are shown by detailed race/ethnicity in Table 3. We estimated both age-adjusted and multivariable-adjusted HRs and found that the multivariable adjusted HRs were higher for NHPI (HR, 1.07; 95% CI, 0.87-1.31) and AIAN (HR, 1.05; 95% CI, 0.87-1.26) female patients compared with NHW female patients. The HR for NHB female patients was 0.94 (95% CI, 0.89-0.99) and the HR for Hispanic female patients was 0.89 (95% CI, 0.85-0.93), exhibiting lower mortality compared with NHW female patients. Among AANHPI ethnic groups, Chinese female patients had an adjusted HR of 0.82 (95% CI, 0.76-0.88). In addition, Filipino, Korean, and Vietnamese female patients had lower mortality with HRs of 0.80 (95% CI, 0.74-0.87), 0.82 (95% CI, 0.72-0.94), and 0.76 (95% CI, 0.68-0.84), respectively, relative to NHW female patients.

**Table 3:** HRs for All-Cause Mortality According to Race/Ethnicity: NSCLC in Female Patients Diagnosed From 2014 to 2019 in California

Race/Ethnicity	No. of Cases	No. of Deaths (All-Cause)	HR Age Adjusted^a^	Lower 95% CI	Upper 95% CI	HR Multivariable Adjusted^b^	Lower 95% CI	Upper 95% CI
Non-Hispanic White	18,118	12,435	1.0	NA	1.0	NA	NA	NA
Non-Hispanic Black	2,315	1,679	1.06	1.01	1.11	0.94	0.89	0.99
Hispanic	3,874	2,550	0.91	0.87	0.95	0.89	0.85	0.93
Chinese	1,441	820	0.64	0.60	0.68	0.82	0.76	0.88
Japanese	285	197	0.83	0.73	0.95	0.90	0.79	1.03
Filipino	1,031	587	0.71	0.65	0.77	0.80	0.74	0.87
Korean	279	174	0.69	0.60	0.78	0.82	0.72	0.94
Vietnamese	624	379	0.65	0.58	0.71	0.76	0.68	0.84
Southeast Asian^c^	165	110	0.84	0.71	1.01	0.91	0.75	1.10
South Asian^d^	160	81	0.66	0.53	0.84	0.79	0.62	1.00
Other Asian^e^	189	111	0.72	0.60	0.85	0.89	0.75	1.06
Native Hawaiian, Pacific Islander	152	104	1.05	0.86	1.28	1.07	0.87	1.31
American Indian, Alaska Native	221	167	1.08	0.91	1.29	1.05	0.87	1.26

HR = hazard ratio; NA = not applicable; NSCLC = non-small cell lung cancer.

a Adjusted for age at diagnosis (continuous) with American Joint Committee on Cancer stage, radiation, and chemotherapy as strata.

b Multivariable adjusted for age at diagnosis (continuous), histology, surgery, insurance status, marital status, tobacco smoking history (imputed for missing), ever seen at National Cancer Institute-designated cancer center for diagnosis and/or treatment (yes, no), Charlson Comorbidity Index score, and neighborhood socioeconomic status (quintile) with American Joint Committee on Cancer stage, radiation, and chemotherapy as strata.

c Lao, Hmong, Kampuchean, Cambodian, and Thai.

d Indian, Pakistani, Sri Lankan, and Bangladeshi.

e Bhutanese, Nepalese, Sikkimese, Burmese, Indonesian, and other Asian, Asian not otherwise specified.

The adjusted HRs for all-cause mortality among male indviduals across race and ethnicity are presented in Table 4. Compared with NHW male individuals, Japanese (HR, 1.14; 95% CI, 0.99-1.31) and AIAN (HR, 1.15; 95% CI, 0.96-1.38) male patients had increased mortality. NHB male patients had an HR of 0.91 (95% CI, 0.86-0.95), which was comparable to Hispanic male patients who had an HR of 0.96 (95% CI, 0.92-1.00). Among AANHPI ethnic groups, other Asian (HR, 0.77; 95% CI, 0.65-0.92) and Vietnamese (HR, 0.78; 95% CI, 0.72-0.84) male individuals had the lowest mortality, relative to NHW male individuals.

**Table 4:** HRs for All-Cause Mortality According to Race/Ethnicity: NSCLC in Male Individuals Diagnosed From 2014 to 2019 in California

Race/Ethnicity	No. of Cases	No. of Deaths (All-Cause)	HR Age Adjusted^a^	Lower 95% CI	Upper 95% CI	HR Multivariable Adjusted^b^	Lower 95% CI	Upper 95% CI
Non-Hispanic White	17,802	13,680	1.0	NA	NA	1.0	NA	NA
Non-Hispanic Black	2,333	1,873	1.03	0.98	1.08	0.91	0.86	0.95
Hispanic	4,034	3,160	0.99	0.95	1.03	0.96	0.92	1.00
Chinese	1,557	1,033	0.68	0.64	0.72	0.79	0.74	0.84
Japanese	245	200	1.03	0.90	1.19	1.14	0.99	1.31
Filipino	1,219	896	0.85	0.80	0.90	0.87	0.81	0.93
Korean	399	293	0.79	0.71	0.89	0.86	0.76	0.97
Vietnamese	950	658	0.73	0.68	0.79	0.78	0.72	0.84
Southeast Asian^c^	176	142	0.89	0.77	1.04	0.90	0.77	1.05
South Asian^d^	261	180	0.79	0.69	0.91	0.85	0.74	0.98
Other Asian^e^	168	118	0.70	0.59	0.82	0.77	0.65	0.92
Native Hawaiian, Pacific Islander	158	119	0.89	0.77	1.04	0.91	0.79	1.06
American Indian, Alaska Native	197	159	1.25	1.03	1.52	1.15	0.96	1.38

HR = hazard ratio; NA = not applicable; NSCLC = non-small cell lung cancer.

a Adjusted for age at diagnosis (continuous) with American Joint Committee on Cancer stage, radiation, and chemotherapy as strata.

b Multivariable adjusted for age at diagnosis (continuous), histology, surgery, insurance status, marital status, tobacco smoking history (imputed for missing), ever seen at National Cancer Institute-designated cancer center for diagnosis and/or treatment (yes, no), Charlson Comorbidity Index score, and neighborhood socioeconomic status (quintile) with American Joint Committee on Cancer stage, radiation, and chemotherapy as strata.

c Lao, Hmong, Kampuchean, Cambodian, and Thai.

d Indian, Pakistani, Sri Lankan, and Bangladeshi.

e Bhutanese, Nepalese, Sikkimese, Burmese, Indonesian, and other Asian, Asian not otherwise specified.

To further examine mortality differences according to race and ethnicity, we assessed differences by smoking history (Table 5). Among female patients who never smoked, NHPI female patients had the highest HR at 1.33 (95% CI, 1.02-1.73), whereas Vietnamese female patients had the lowest HR at 0.80 (95% CI, 0.69-0.92), relative to NHW female patients. Among female patients who currently or formerly smoked, NHPI female patients had the highest mortality with an HR of 1.05 (95% CI, 0.76-1.43); Filipino female patients had the lowest mortality with an HR of 0.78 (95% CI, 0.70-0.88).

**Table 5:** HRs for NSCLC in Female Patients According to Tobacco Smoking History (Using Imputed Values for Missing): All-Cause Mortality, Diagnosis From 2014 to 2019 in California

Race/Ethnicity	Never Smoked					Currently or Formerly Smoked		
	No. of Cases	No. of Deaths, All-Cause	HR^a^	95% CI	No. of Cases	No. of Deaths, All-Cause	HR^a^	95% CI
Non-Hispanic White	2,658	1,439	1.0	NA	NA	15,460	10,996	1.0
Non-Hispanic Black	326	200	1.01	0.86-1.18	1,989	1,479	0.92	0.87-0.98
Hispanic	1,248	728	0.98	0.89-1.08	2,626	1,822	0.89	0.84-0.93
Chinese	984	504	0.92	0.83-1.03	457	316	0.83	0.75-0.92
Japanese	85	48	0.98	0.71-1.35	200	149	0.91	0.80-1.04
Filipino	544	290	0.90	0.80-1.02	487	297	0.78	0.70-0.88
Korean	172	105	0.95	0.78-1.14	107	69	0.81	0.64-1.01
Vietnamese	417	228	0.80	0.69-0.92	207	151	0.84	0.72-0.99
Southeast Asian^b^	111	71	1.06	0.84-1.33	54	39	0.92	0.66-1.29
South Asian^c^	129	58	0.87	0.66-1.16	31	23	1.02	0.63-1.62
Other Asian^d^	143	84	1.10	0.89-1.36	46	27	0.87	0.61-1.24
Native Hawaiian, Pacific Islander	88	60	1.33	1.02-1.73	64	44	1.05	0.76-1.43
American Indian, Alaska Native	73	51	1.27	0.94-1.70	148	116	1.04	0.84-1.29

HR = hazard ratio; NA = not applicable; NSCLC = non-small cell lung cancer.

a Multivariable HRs adjusted for age at diagnosis (continuous), histology, surgery, insurance status, marital status, ever seen at National Cancer Institute-designated cancer center for diagnosis and/or treatment (yes, no), Charlson Comorbidity Index score, and neighborhood socioeconomic status (quintile) with American Joint Committee on Cancer stage, radiation, and chemotherapy as strata.

b Lao, Hmong, Kampuchean, Cambodian, and Thai.

c Indian, Pakistani, Sri Lankan, and Bangladeshi.

d Bhutanese, Nepalese, Sikkimese, Burmese, Indonesian, and other Asian, Asian not otherwise specified.

Among male patients who never smoked (Table 6), Japanese male patients had the highest mortality with an HR of 1.72 (95% CI 1.14-2.59) compared with NHW male patients who never smoked. Among male patients who never smoked, Hispanic male patients had a higher mortality with an HR of 1.07 (95% CI, 0.92-1.23); NHB male patients had an HR of 0.86 (95% CI, 0.70-1.05). Among male patients who currently or formerly smoked, Chinese and Filipino male patients had a significantly lower HR of 0.78 (95% CI, 0.73-0.84) and 0.85 (95% CI, 0.79-0.91), respectively. In contrast, Japanese male patients who smoked had higher mortality (HR, 1.09; 95% CI, 0.94-1.27).

**Table 6:** HRs for NSCLC All-Cause Mortality in Male Patients According to Tobacco Smoking History (Using Imputed Values for Missing): Diagnosed From 2014 to 2019 in California

Race/Ethnicity	Never Smoked				Currently or Formerly Smoked			
	No. of Cases	No. of Deaths, All-Cause	HR^a^	95% CI	No. of Cases	No. of Deaths, All-Cause	HR^a^	95% CI
Non-Hispanic White	1,344	876	1.0	NA	16,458	12,804	1.0	NA
Non-Hispanic Black	126	95	0.86	0.70-1.05	2,207	1,778	0.91	0.86-0.96
Hispanic	473	335	1.07	0.92-1.23	3,561	2,825	0.94	0.90-0.98
Chinese	325	199	0.83	0.71-0.97	1,232	834	0.78	0.73-0.84
Japanese	26	20	1.72	1.14-2.59	219	180	1.09	0.94-1.27
Filipino	105	78	1.11	0.87-1.42	1,114	818	0.85	0.79-0.91
Korean	44	32	1.05	0.75-1.48	355	261	0.85	0.75-0.96
Vietnamese	89	66	0.88	0.68-1.15	861	592	0.77	0.70-0.83
Southeast Asian^b^	34	25	0.86	0.57-1.29	142	117	0.89	0.75-1.05
South Asian^c^	62	41	0.86	0.58-1.25	199	139	0.84	0.71-0.98
Other Asian^d^	26	17	0.77	0.54-1.09	142	101	0.77	0.64-0.93
Native Hawaiian, Pacific Islander	15	< 11	Too few		143	110	0.90	0.77-1.05
American Indian/Alaska Native	< 11	< 11	Too few		187	152	1.15	0.96-1.39

HR = hazard ratio; NA = not applicable; NSCLC = non-small cell lung cancer.

a Multivariable HRs adjusted for age at diagnosis (continuous), histology, surgery, insurance status, marital status, ever seen at National Cancer Institute-designated cancer center for diagnosis and/or treatment (yes, no), Charlson Comorbidity Index score, and neighborhood socioeconomic status (quintile) with American Joint Committee on Cancer stage, radiation, and chemotherapy as strata.

b Lao, Hmong, Kampuchean, Cambodian, and Thai.

c Indian, Pakistani, Sri Lankan, and Bangladeshi.

d Bhutanese, Nepalese, Sikkimese, Burmese, Indonesian, and other Asian, Asian not otherwise specified.

As a sensitivity analysis to assess potential biases in including imputed data on smoking status among the 31% of cases with unknown or missing data, we conducted multivariable modeling analyses that were restricted to only patients with known smoking history (e-Tables 2 and 3). No major differences were observed from the HRs calculated using imputed smoking status.

## Discussion

This population-based study assessed survival patterns among detailed racial and ethnic groups, adjusted for and stratified according to smoking status, leveraging newly available smoking information in our cancer registry. Specifically, in a state-wide analysis of racial/ethnic patterns in survival among NSCLC cases, we found that most of the racial and ethnic groups examined had lower mortality relative to NHW patients, with the noteworthy exception of Japanese male patients, NHPI female patients, and AIAN male and female patients. When cases were stratified according to smoking status, the same general differences were observed by race/ethnicity, although higher mortality was observed in Japanese male patients who had never smoked compared with NHW male patients. However, this finding was somewhat limited by sample sizes.

An earlier study using California Cancer Registry data found that Japanese American individuals with NSCLC had higher mortality overall compared with Chinese American individuals.^26^ In the current analysis of California Cancer Registry data, although we did not directly compare across AANHPI ethnic groups, we also noted that Japanese American male individuals seemed to have higher mortality than Chinese American individuals and other Asian American groups. Our findings of differences in mortality patterns in disaggregated Asian ethnic groups are not surprising given that the AANHPI population as a singular group comprises ethnicities from > 40 countries that are distinctly unique in many different ways.^27^

In addition, many prior studies investigating mortality in AANHPI individuals diagnosed with NSCLC were limited because they were based on aggregated ethnicity data, lack of granularity of smoking status documentation, or included only patients with advanced stage or metastatic disease.^28^, ^29^, ^30^, ^31^, ^32^, ^33^ Due to cancer being the leading cause of death in most AANHPI groups, it is extremely important to understand the unique factors behind lung cancer survival in distinct AANHPI ethnic groups,^34^ including assessing the role of health care access and other social drivers, details on smoking and other health behaviors, and information on genetic/genomic, biomarker, and targeted treatments.^35^

Lung cancer among people in California who never smoked is a continued concern, with most estimates of lung cancer cases among AANHPI women who never smoked at ≥ 50% followed by 25% in Hispanic individuals.^36^^,^^37^ Accounting for smoking status is particularly critical given distinct differences in NSCLC diagnosed among patients who do not smoke, which are enriched for epidermal growth factor receptor (EGFR) and anaplastic lymphoma kinase (ALK) mutations, and is more commonly seen among female individuals and AANHPI ethnic groups; in fact, lung cancer among people who do not smoke has been considered as a different disease than that seen among people who do smoke.^38^^,^^39^

Many studies from the United States and Asia have reported lower NSCLC mortality among Asian American inidividuals who do not smoke.^36^^,^^40^^,^^41^ Hu et al^42^ examined 4,552 patients with advanced-stage NSCLC from a large tertiary hospital in China. They found that both male and female Chinese patients who never smoked had lower overall mortality compared with their counterparts with a previous smoking history, and it was never-smoking status that had the most sizable association with overall mortality, not sex. We found lower overall mortality in most Asian American ethnic subgroups who have never smoked similar to previous studies reported, except for our findings of higher mortality in Japanese male individuals who have never smoked. Interestingly, Kawaguchi et al^40^ compared 2 large cohorts of patients with NSCLC from Japan and the United States and found lower mortality for Japanese patients who have never smoked compared with their NHW counterparts. However, these Japanese patients who have never smoked resided in Japan compared with NHW patients who have never smoked in the United States, and after multivariable regression analysis, Japanese female patients who have never smoked had lower mortality compared with Japanese male patients; this possibly infers that the improved prognosis seen in Japanese patients with NSCLC was driven by Japanese female patients who have never smoked.

The current results showed that Hispanic female patients who currently or formerly smoked had lower mortality compared with NHW female patients (HR, 0.89; 95% CI, 0.84-0.93). No mortality difference was seen between Hispanic and NHW female patients who never smoked (HR, 0.98; 95% CI, 0.89-1.08). Our finding of lower mortality for Hispanic female patients who currently smoke or smoked in the past is consistent with a report evaluating 172,398 patients with NSCLC from the SEER database that found Hispanic patients had significantly lower mortality with an HR of 0.85 (95% CI, 0.83-0.87) compared with NHW patients.^43^ However, this large database study did not account for smoking status. More recently, a large urban academic medical center reported lower mortality specifically for Hispanic female patients with NSCLC who ever smoked (HR, 0.72; 95% CI, 0.57-0.90), thus adding to the evidence that the lower mortality in Hispanic female patients is not solely related to smoking status.^44^ Miao et al^45^ reviewed and summarized the multitude of factors that may be contributing to the lower mortality in Hispanic patients with NSCLC. They identified genetic variation, smoking patterns, cultural influences, and comorbidities as factors that may at least partially explain some of the lower mortality.

In this analysis of the California Cancer Registry data, we found lower mortality in NHB patients compared with NHW patients with lung cancer once we adjusted for clinical and demographic factors. This finding is not entirely inconsistent with SEER 22 data (national SEER data from 22 registries), which showed marginally lower 5-year relative survival for NHB patients at 20.1 (95% CI, 19.4-20.8) relative to NHW patients at 22.6 (95% CI, 22.3-22.9) for patients diagnosed from 2012 to 2020.^46^ The difference is a bit more pronounced within California (19.9 and 23.0 for NHB and NHW individuals, respectively). Age adjustment could reasonably explain these modest survival differences. Other studies have also shown lower mortality in NHB patients compared with NHW patients with NSCLC after adjusting for stage and various socioeconomic factors.^7^, ^8^, ^9^ Furthermore, Jones et al^47^ found no difference, after adjusting for stage and treatment, in mortality in Black and White patients with NSCLC from the Southern Community Cohort study (SCCS) based on 12 southern states. This adds more evidence to the notion that Black patients with appropriate early detection and access to care may not experience as inferior lung cancer survival as compared with other ethnicities. We encourage future lung cancer studies on racial and ethnic differences to include representative data sets that include detailed sociodemographic factors to inform the most optimal targeted intervention strategies for reducing any future potential disparities in survival.

The current study does have several strengths, most notably investigating a disaggregated AANHPI cohort with NSCLC with stratification according to sex and smoking history, but also including the largest statewide and most diverse real-world population cohort as it pertains to multiple racial and ethnic groups. There are also some limitations to this study. First, although smoking information was recently made available in California Cancer Registry data, it was nonetheless missing for 31% of our cohort. To mitigate this issue, smoking information was imputed for those cases with missing tobacco smoking history; thus, the smoking information used in our analysis, although generally robust, is considered an estimate. We conducted a large number of statistical tests, and there is the possibility of false-positive associations. Lastly, although the importance of biomarker status for NSCLC has emerged recently as a potential factor contributing to overall mortality in patients with NSCLC, we could not account for biomarker testing in our database.

## Interpretation

The current study reports overall mortality differences in NSCLC according to race and ethnicity when stratifying on sex, smoking status, and sociodemographic factors. These findings, which are based on the largest statewide cancer registry that includes diverse racial/ethnic populations in the United States, are generally powered to provide significant survival data across multiple racial/ethnic groups. In addition, a novel aspect to this study was the reporting of NSCLC mortality differences among 8 distinct AANHPI populations when stratified by sex and smoking history. Future research on factors affecting NSCLC mortality should continue to investigate access to care and structural and social drivers of health, along with tumor biomarker information, as it relates to each distinct racial and ethnic group.

## Funding/Support

The collection of cancer incidence data used in this study was supported by the 10.13039/100005002California Department of Public Health pursuant to California Health and Safety Code Section 103885; Centers for Disease Control and Prevention’s National Program of Cancer Registries, under cooperative agreement 1NU58DP007156; the NCI’s SEER Program under contract HHSN261201800032I awarded to the University of California, San Francisco, contract HHSN261201800015I awarded to the University of Southern California, and contract HHSN261201800009I awarded to the Public Health Institute, Cancer Registry of Greater California.

## Financial/Nonfinancial Disclosures

None declared.

## Supplementary Data

e-Online Data

e-Online Data

e-Online Data

e-Online Data
