# Supplementary material for: Non-Small Cell Lung Cancer Survival by Race and Ethnicity in California, 2014-2019 Differences by Sex and Smoking History
Source: CHEST Pulm. 2025 Jun 20;3(4):100190. doi: 10.1016/j.chpulm.2025.100190 (PMC13417799; doi:10.1016/j.chpulm.2025.100190)
Supplement: e-Online Data [file mmc1.docx]

**e-Table 3. Hazard ratios for all-cause mortality by race/ethnicity, Non-Small Cell Lung Cancer in Males diagnosed 2014-2019 in California, among cases with known smoking history only.**

| Race/Ethnicity | Hazard Ratio  multivariable adjusted^**^ | Lower 95% CI | Upper CI |
| --- | --- | --- | --- |
| Non-Hispanic White | 1.0 (ref) |  |  |
| Non-Hispanic Black | 0.918 | 0.857 | 0.983 |
| Hispanic | 0.922 | 0.872 | 0.975 |
| Chinese | 0.840 | 0.768 | 0.920 |
| Japanese | 0.911 | 0.783 | 1.061 |
| Filipino | 0.808 | 0.719 | 0.908 |
| Korean | 0.850 | 0.721 | 1.001 |
| Vietnamese | 0.730 | 0.637 | 0.837 |
| Southeast Asian ^a^ | 0.891 | 0.720 | 1.103 |
| South Asian ^b^ | 0.853 | 0.644 | 1.129 |
| Other Asian ^c^ | 0.915 | 0.735 | 1.138 |
| Native Hawaiian, Pacific Islander | 1.026 | 0.816 | 1.290 |
| American Indian, Alaska Native | 1.036 | 0.843 | 1.273 |

**^a^** Lao, Hmong, Kampuchean, Cambodian, and Thai

**^b^** Indian, Pakistani, Sri Lankan, and Bangladeshi

^c^ Bhutanese, Nepalese, Sikkimese, Burmese, Indonesian, and Other Asian, Asian not otherwise specified

^*^Multivariable adjusted for age at diagnosis (continuous), histology, surgery, insurance status, marital status, tobacco smoking history (not imputed for missing, missing were excluded), ever seen at National Cancer Institute designated cancer center for diagnosis and/or treatment (yes, no), Charlson Comorbidity score, and neighborhood Socioeconomic status (quintile) with AJCC stage, radiation and chemotherapy as strata.
