# Supplementary material for: Non-Small Cell Lung Cancer Survival by Race and Ethnicity in California, 2014-2019 Differences by Sex and Smoking History
Source: CHEST Pulm. 2025 Jun 20;3(4):100190. doi: 10.1016/j.chpulm.2025.100190 (PMC13417799; doi:10.1016/j.chpulm.2025.100190)
Supplement: e-Online Data [file mmc3.docx]

| Supplemental Figure 1. | Percent Surviving, females | | | | | | |
| --- | --- | --- | --- | --- | --- | --- | --- |
| Race/ethnicity | Year 1 | Year 2 | Year 3 | Year 4 | Year 5 | Year 6 | Year 7 |
| Non-Hispanic White | 57.5% | 45.0% | 38.1% | 33.3% | 29.5% | 26.3% | 24.3% |
| Non-Hispanic Black | 53.6% | 40.8% | 33.9% | 29.4% | 25.2% | 22.3% | 20.2% |
| Hispanic | 60.0% | 47.4% | 39.6% | 33.6% | 30.0% | 27.1% | 24.2% |
| Chinese | 73.1% | 59.6% | 50.5% | 44.1% | 37.9% | 34.0% | 30.5% |
| Japanese | 58.8% | 43.6% | 36.5% | 32.0% | 29.6% | 26.4% | 25.2% |
| Filipino | 71.2% | 60.5% | 50.7% | 45.3% | 39.5% | 33.6% | 28.7% |
| Korean | 72.6% | 54.4% | 43.5% | 38.2% | 31.7% | 30.8% | 25.6% |
| Vietnamese | 73.9% | 60.1% | 49.0% | 41.7% | 34.8% | 31.7% | 25.4% |
| Southeast Asian | 60.6% | 47.2% | 39.6% | 33.4% | 28.7% | 23.0% | 23.0% |
| South Asian | 73.8% | 63.0% | 56.9% | 51.9% | 42.4% | 35.9% | 32.7% |
| Other Asian | 70.3% | 58.0% | 47.2% | 40.3% | 34.7% | 34.7% | 29.4% |
| Pacific Islander | 59.4% | 41.5% | 34.3% | 31.5% | 27.0% | 22.4% | 22.4% |
| American Indian/Alaska Native | 54.3% | 38.2% | 30.3% | 26.0% | 23.3% | 21.7% | 19.1% |


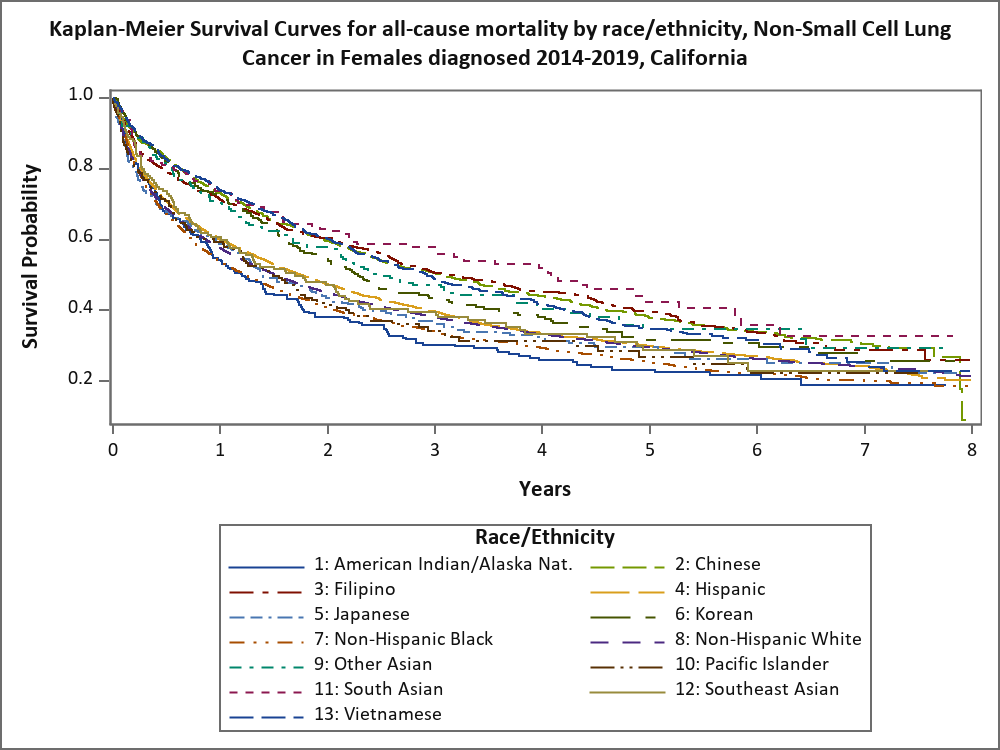


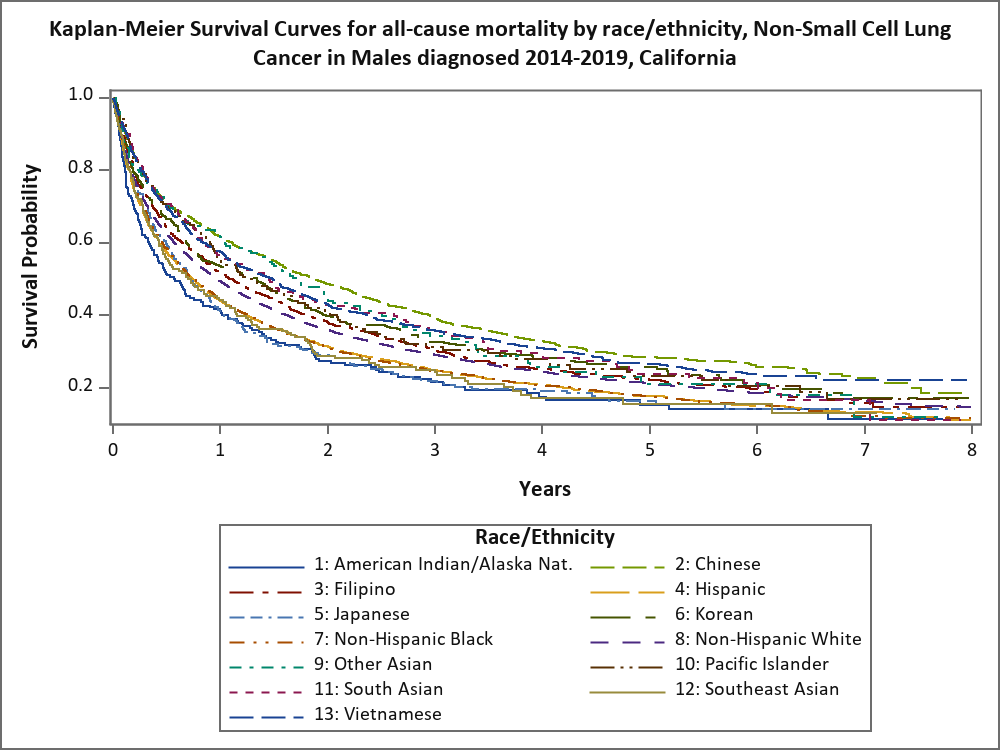


| Supplemental Figure 2. | Percent Surviving, males | | | | | | |
| --- | --- | --- | --- | --- | --- | --- | --- |
| Race/ethnicity | Year 1 | Year 2 | Year 3 | Year 4 | Year 5 | Year 6 | Year 7 |
| Non-Hispanic White | 49.4% | 36.1% | 29.2% | 24.4% | 21.3% | 18.6% | 16.6% |
| Non-Hispanic Black | 44.2% | 31.3% | 24.8% | 20.8% | 17.7% | 15.5% | 12.2% |
| Hispanic | 44.1% | 31.5% | 24.9% | 20.9% | 17.7% | 15.2% | 13.5% |
| Chinese | 61.7% | 48.7% | 39.4% | 33.0% | 28.6% | 25.9% | 22.8% |
| Japanese | 41.2% | 29.2% | 22.0% | 19.2% | 16.4% | 14.2% | 14.2% |
| Filipino | 51.8% | 38.1% | 30.3% | 25.1% | 22.3% | 19.6% | 15.9% |
| Korean | 53.7% | 39.8% | 32.7% | 28.3% | 25.8% | 21.5% | 17.3% |
| Vietnamese | 57.6% | 43.0% | 35.9% | 31.1% | 26.5% | 23.8% | 22.2% |
| Southeast Asian | 44.3% | 28.9% | 24.3% | 17.1% | 15.6% | 15.6% | 13.0% |
| South Asian | 56.4% | 43.0% | 36.1% | 29.0% | 23.9% | 21.4% | 16.6% |
| Other Asian | 61.6% | 44.7% | 34.4% | 25.7% | 21.0% | 21.0% | 12.0% |
| Pacific Islander | 55.8% | 40.9% | 31.3% | 28.0% | 23.4% | 20.7% | 16.9% |
| American Indian/Alaska Native | 41.2% | 27.5% | 21.7% | 17.5% | 15.4% | 14.2% | 11.4% |

| Supplemental Figure 3. | Percent Surviving, non-smoking females | | | | | |  |
| --- | --- | --- | --- | --- | --- | --- | --- |
| Race/ethnicity | Year 1 | Year 2 | Year 3 | Year 4 | Year 5 | Year 6 | Year 7 |
| Non-Hispanic White | 68.5% | 56.6% | 48.8% | 42.6% | 38.2% | 34.7% | 32.1% |
| Non-Hispanic Black | 63.3% | 49.4% | 43.0% | 37.2% | 32.9% | 26.0% | 24.2% |
| Hispanic | 65.3% | 52.4% | 43.3% | 36.3% | 31.3% | 27.8% | 23.9% |
| Chinese | 76.1% | 63.0% | 53.0% | 47.2% | 40.3% | 37.0% | 33.4% |
| Japanese | 67.6% | 53.2% | 45.5% | 35.5% | 32.9% | 28.8% | 28.8% |
| Filipino | 75.0% | 63.1% | 51.8% | 46.5% | 39.2% | 35.0% | 31.2% |
| Korean | 73.6% | 54.4% | 41.1% | 36.2% | 29.4% | 27.7% | 17.8% |
| Vietnamese | 77.1% | 64.5% | 52.5% | 44.3% | 36.4% | 33.6% | 25.3% |
| Southeast Asian | 66.0% | 50.6% | 42.0% | 33.5% | 31.3% | 22.3% | 22.3% |
| South Asian | 77.1% | 63.9% | 55.3% | 49.9% | 41.6% | 30.2% | 24.2% |
| Other Asian | 75.0% | 59.5% | 46.4% | 37.2% | 32.9% | 32.9% | 28.8% |
| Pacific Islander | 62.6% | 43.1% | 37.0% | 34.7% | 27.8% | 16.7% | 16.7% |
| American Indian/Alaska Nat. | 61.9% | 52.4% | 38.1% | Ns too small | | | |


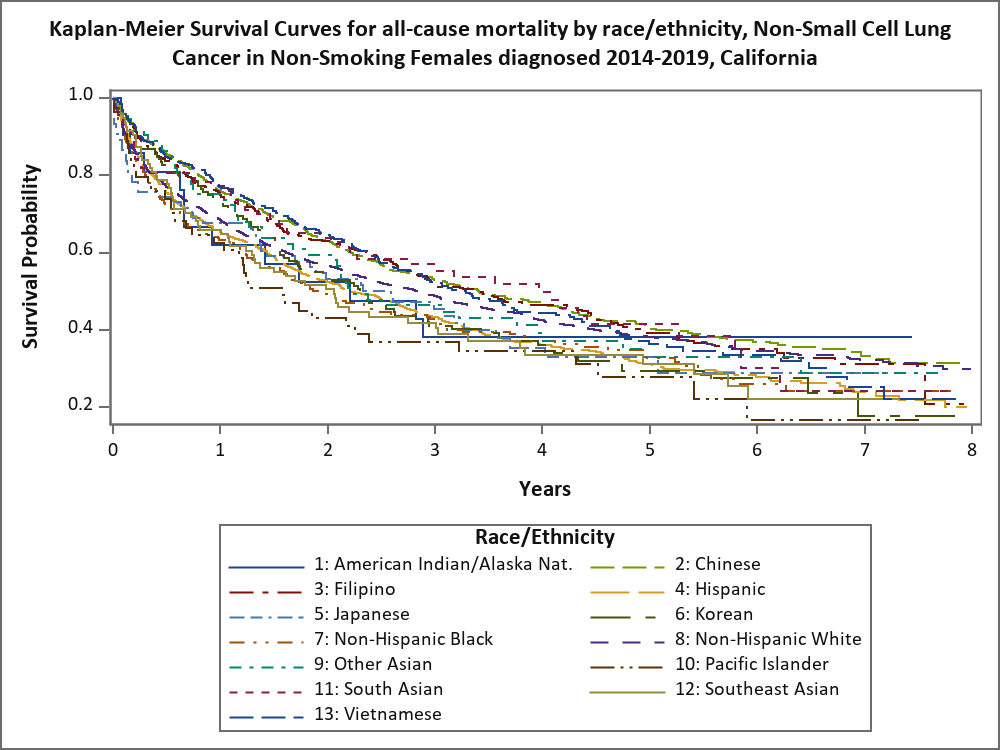


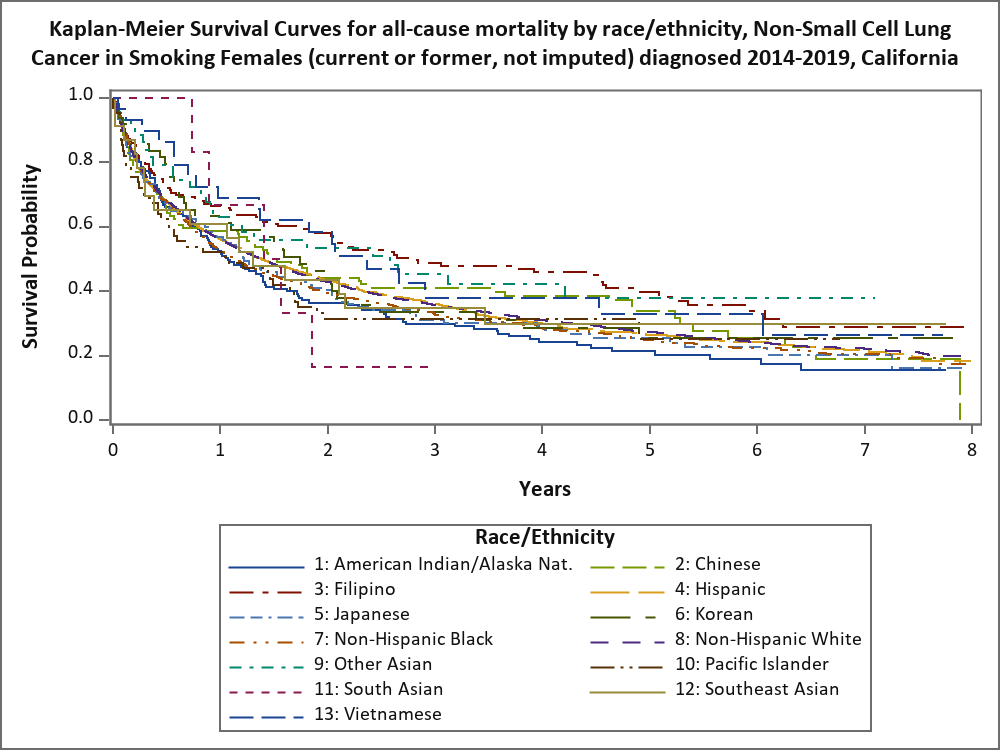


| Supplemental Figure 4. | Percent Surviving, females, ever smoking | | | | | | |  |  |  |  |  |
| --- | --- | --- | --- | --- | --- | --- | --- | --- | --- | --- | --- | --- |
| Race/ethnicity | Year 1 | Year 2 | Year 3 | Year 4 | Year 5 | Year 6 | Year 7 |  |  |  |  |  |
| Non-Hispanic White | 56.1% | 42.9% | 35.9% | 31.1% | 27.3% | 24.1% | 22.3% |  |  |  |  |  |
| Non-Hispanic Black | 52.5% | 39.6% | 32.9% | 28.6% | 24.9% | 22.5% | 20.5% |  |  |  |  |  |
| Hispanic | 56.1% | 43.4% | 36.0% | 30.0% | 26.5% | 24.3% | 21.8% |  |  |  |  |  |
| Chinese | 58.7% | 44.2% | 41.2% | 38.7% | 33.8% | 25.7% | 19.0% |  |  |  |  |  |
| Japanese | 56.8% | 40.3% | 31.3% | 28.1% | 25.5% | 22.7% | 20.2% |  |  |  |  |  |
| Filipino | 66.5% | 58.0% | 48.8% | 46.2% | 39.7% | 33.8% | 28.9% |  |  |  |  |  |
| Korean | 63.3% | 46.4% | 33.6% | 28.8% | 25.6% | 25.6% | 25.6% |  |  |  |  |  |
| Vietnamese | 69.0% | 58.4% | 37.9% | 37.9% | 33.1% | 33.1% | 26.5% |  |  |  |  |  |
| Southeast Asian | 60.9% | 43.5% | 34.8% | 29.8% | Ns too small | | |  |  |  |  |  |
| South Asian | 66.7% | Ns too small | | | | | |  |  |  |  | . |
| Other Asian | 62.9% | 53.5% | 45.5% | 42.2% | 38.0% | 38.0% | 38.0% |  |  |  |  |  |
| Pacific Islander | 52.2% | 31.6% | 31.6% | 31.6% | 25.3% | Ns too small | |  |  |  |  |  |
| American Indian/Alaska Nat | 52.4% | 36.5% | 29.9% | 24.4% | 21.4% | 19.1% | 15.5% |  |  |  |  |  |


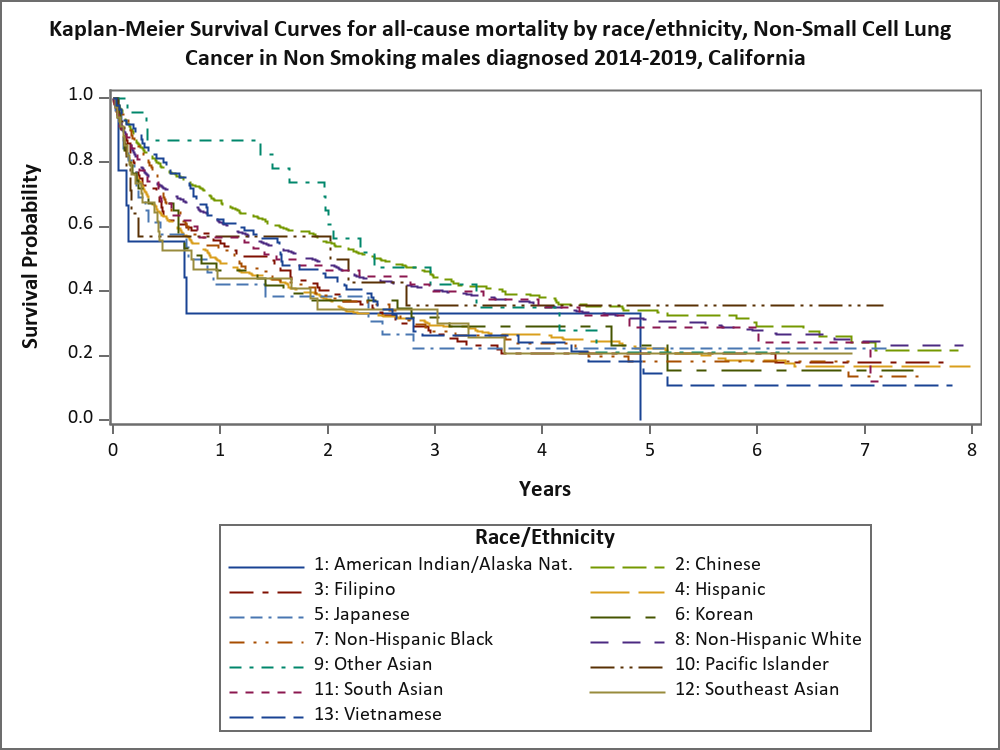


| Supplemental Figure 5. | Percent Surviving, non-smoking males | | | | | | |
| --- | --- | --- | --- | --- | --- | --- | --- |
| Race/ethnicity | Year 1 | Year 2 | Year 3 | Year 4 | Year 5 | Year 6 | Year 7 |
| Non-Hispanic White | 61.7% | 48.6% | 40.5% | 35.7% | 31.0% | 27.9% | 24.5% |
| Non-Hispanic Black | 52.9% | 38.8% | 27.8% | 24.0% | 18.5% | 18.5% | 13.9% |
| Hispanic | 48.9% | 37.5% | 29.5% | 26.4% | 22.4% | 18.7% | 16.8% |
| Chinese | 68.2% | 55.4% | 44.6% | 38.3% | 34.3% | 29.3% | 24.2% |
| Japanese | 42.3% | 38.5% | 22.4% | 22.4% | 22.4% | 22.4% | 22.4% |
| Filipino | 55.0% | 40.4% | 26.8% | 20.9% | 20.9% | 20.9% | 18.3% |
| Korean | 46.5% | 37.2% | 32.1% | 29.4% | 23.5% | 15.7% | 15.7% |
| Vietnamese | 62.5% | 44.4% | 26.4% | 24.2% | 14.8% | 11.1% | 11.1% |
| Southeast Asian | 44.1% | 34.7% | 34.7% | 20.8% | 20.8% | Ns too small | |
| South Asian | 56.9% | 46.5% | 40.1% | 35.2% | 29.0% | 29.0% | 24.2% |
| Other Asian | 87.0% | 65.2% | 42.2% | 35.1% | 21.1% | Ns too small | |
| Pacific Islander | 57.1% | 57.1% | 35.7% | 35.7% | Ns too small | | |
| American Indian/Alaska Native | 33.3% | 33.3% | Ns too small | | | | |


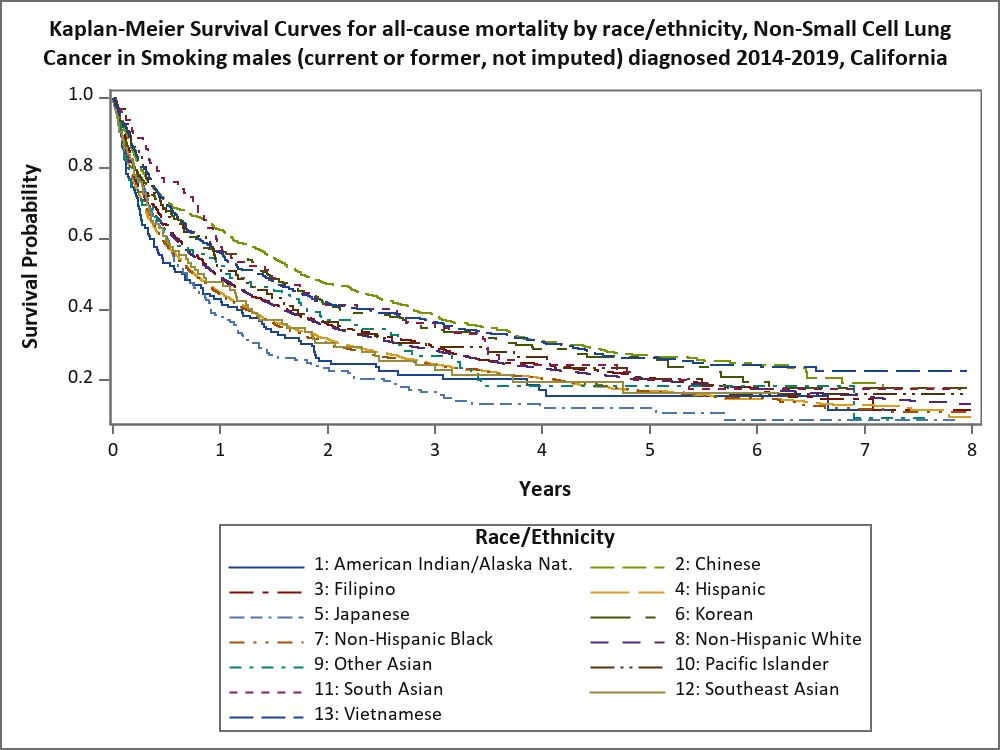


| Supplemental Figure 6. | Percent Surviving, smoking males | | | | | | |
| --- | --- | --- | --- | --- | --- | --- | --- |
| Race/ethnicity | Year 1 | Year 2 | Year 3 | Year 4 | Year 5 | Year 6 | Year 7 |
| Non-Hispanic White | 49.2% | 35.4% | 28.4% | 23.3% | 20.2% | 17.5% | 15.5% |
| Non-Hispanic Black | 44.1% | 30.4% | 24.3% | 20.4% | 16.7% | 14.8% | 11.7% |
| Hispanic | 44.8% | 31.8% | 24.4% | 20.4% | 16.9% | 14.6% | 13.1% |
| Chinese | 62.6% | 47.3% | 38.4% | 31.0% | 27.0% | 24.2% | 19.3% |
| Japanese | 37.8% | 23.4% | 16.5% | 12.1% | 12.1% | 8.6% | 8.6% |
| Filipino | 49.1% | 35.8% | 29.0% | 24.2% | 20.7% | 17.6% | 14.6% |
| Korean | 56.6% | 41.3% | 34.3% | 28.7% | 26.6% | 19.6% | 17.8% |
| Vietnamese | 56.1% | 41.9% | 36.3% | 30.9% | 26.3% | 24.4% | 22.7% |
| Southeast Asian | 47.8% | 30.4% | 22.8% | 19.5% | 16.3% | Ns too small | |
| South Asian | 58.9% | 41.3% | 36.2% | 24.2% | 20.1% | 17.6% | 17.6% |
| Other Asian | 52.3% | 37.0% | 26.8% | 18.4% | 18.4% | 18.4% | 9.2% |
| Pacific Islander | 52.1% | 36.6% | 29.3% | 26.4% | 20.2% | 16.2% | 16.2% |
| American Indian/Alaska Native | 43.0% | 25.4% | 21.6% | 17.1% | 15.5% | 15.5% | 11.7% |
