# Supplementary material for: Non-Small Cell Lung Cancer Survival by Race and Ethnicity in California, 2014-2019 Differences by Sex and Smoking History
Source: CHEST Pulm. 2025 Jun 20;3(4):100190. doi: 10.1016/j.chpulm.2025.100190 (PMC13417799; doi:10.1016/j.chpulm.2025.100190)
Supplement: e-Online Data [file mmc4.docx]

| **e-Table 1. Non-small cell lung cancer (NSCLC) Cases Characteristics by Smoking Status, California, Diagnosed 2014-19** | | | | | | |  |  |  |
| --- | --- | --- | --- | --- | --- | --- | --- | --- | --- |
|  | **Smoking history** | | | | | | | | |
|  | **Never used** | | | **Current/Former user** | | | **Unknown** | | |
|  | **N** | **row %** | **col %** | **N** | **row %** | **col %** | **N** | **row %** | **col %** |
| **All** | 8364 | 14.3 | 100.0 | 31308 | 53.7 | 100.0 | 18681 | 32.0 | 100.0 |
| **sex** | 2632 | 8.9 | 31.5 | 17307 | 58.7 | 55.3 | 9560 | 32.4 | 51.2 |
| Male |  |  |  |  |  |  |  |  |  |
| Female | 5732 | 19.9 | 68.5 | 14001 | 48.5 | 44.7 | 9121 | 31.6 | 48.8 |
| **Age category at diagnosis (years)** | 832 | 38.7 | 9.9 | 703 | 32.7 | 2.2 | 617 | 28.7 | 3.3 |
| <50 |  |  |  |  |  |  |  |  |  |
| 50-59 | 1330 | 17.3 | 15.9 | 4068 | 52.9 | 13.0 | 2296 | 29.8 | 12.3 |
| 60-69 | 2316 | 13.0 | 27.7 | 9831 | 55.3 | 31.4 | 5640 | 31.7 | 30.2 |
| 70-79 | 2267 | 11.5 | 27.1 | 11037 | 56.0 | 35.3 | 6399 | 32.5 | 34.3 |
| 80+ | 1619 | 14.7 | 19.4 | 5669 | 51.5 | 18.1 | 3729 | 33.8 | 20.0 |
| **Race/ethnicity** | 3539 | 9.9 | 42.3 | 21791 | 60.7 | 69.6 | 10590 | 29.5 | 56.7 |
| Non-Hispanic White |  |  |  |  |  |  |  |  |  |
| Non-Hispanic Black | 386 | 8.3 | 4.6 | 2567 | 55.2 | 8.2 | 1695 | 36.5 | 9.1 |
| Hispanic | 1544 | 19.5 | 18.5 | 3479 | 44.0 | 11.1 | 2885 | 36.5 | 15.4 |
| Chinese | 1179 | 39.3 | 14.1 | 782 | 26.1 | 2.5 | 1037 | 34.6 | 5.6 |
| Japanese | 100 | 18.9 | 1.2 | 266 | 50.2 | 0.8 | 164 | 30.9 | 0.9 |
| Filipino | 553 | 24.6 | 6.6 | 792 | 35.2 | 2.5 | 905 | 40.2 | 4.8 |
| Korean | 188 | 27.7 | 2.2 | 284 | 41.9 | 0.9 | 206 | 30.4 | 1.1 |
| Vietnamese | 405 | 25.7 | 4.8 | 564 | 35.8 | 1.8 | 605 | 38.4 | 3.2 |
| Southeast Asian | 128 | 37.5 | 1.5 | 115 | 33.7 | 0.4 | 98 | 28.7 | 0.5 |
| South Asian | 147 | 34.9 | 1.8 | 104 | 24.7 | 0.3 | 170 | 40.4 | 0.9 |
| Other Asian | 97 | 27.2 | 1.2 | 131 | 36.7 | 0.4 | 129 | 36.1 | 0.7 |
| Pacific Islander | 68 | 21.9 | 0.8 | 155 | 50.0 | 0.5 | 87 | 28.1 | 0.5 |
| American Indian/Alaska Native | 30 | 7.2 | 0.4 | 278 | 66.5 | 0.9 | 110 | 26.3 | 0.6 |
| **AJCC Stage** | 1860 | 14.4 | 22.2 | 6898 | 53.5 | 22.0 | 4132 | 32.1 | 22.1 |
| I |  |  |  |  |  |  |  |  |  |
| II | 527 | 12.1 | 6.3 | 2469 | 56.7 | 7.9 | 1359 | 31.2 | 7.3 |
| III | 1032 | 9.9 | 12.3 | 6276 | 60.0 | 20.0 | 3144 | 30.1 | 16.8 |
| IV | 4945 | 16.1 | 59.1 | 15665 | 51.1 | 50.0 | 10046 | 32.8 | 53.8 |
| **Histologic type (ICDO-3)** | 663 | 9.2 | 7.9 | 3925 | 54.3 | 12.5 | 2640 | 36.5 | 14.1 |
| unspecified carcinoma |  |  |  |  |  |  |  |  |  |
| large cell + other specified carcinoma | 846 | 17.9 | 10.1 | 2368 | 50.0 | 7.6 | 1524 | 32.2 | 8.2 |
| adenocarcinoma | 6248 | 18.1 | 74.7 | 17331 | 50.1 | 55.4 | 11030 | 31.9 | 59.0 |
| squamous cell | 607 | 5.2 | 7.3 | 7684 | 65.2 | 24.5 | 3487 | 29.6 | 18.7 |
| **Surgery** |  |  |  |  |  |  |  |  |  |
| no | 6061 | 13.6 | 72.5 | 24238 | 54.5 | 77.4 | 14186 | 31.9 | 75.9 |
| yes | 2302 | 16.6 | 27.5 | 7056 | 51.0 | 22.5 | 4472 | 32.3 | 23.9 |
| unknown | <11 |  |  | 13 | 35.1 | 0.0 | 23 | 62.2 | 0.1 |
| **Chemotherapy** | 4225 | 12.4 | 50.5 | 18344 | 53.7 | 58.6 | 11588 | 33.9 | 62.0 |
| no |  |  |  |  |  |  |  |  |  |
| yes | 3989 | 17.2 | 47.7 | 12395 | 53.4 | 39.6 | 6809 | 29.4 | 36.4 |
| unknown | 150 | 15.0 | 1.8 | 569 | 56.7 | 1.8 | 284 | 28.3 | 1.5 |
| **Radiation treatment** | 5859 | 15.3 | 70.1 | 19219 | 50.2 | 61.4 | 13240 | 34.6 | 70.9 |
| no |  |  |  |  |  |  |  |  |  |
| yes | 2504 | 12.5 | 29.9 | 12073 | 60.4 | 38.6 | 5421 | 27.1 | 29.0 |
| unknown | <11 |  |  | 16 | 43.2 | 0.1 | 20 | 54.1 | 0.1 |
| **Charlson comorbidity score** | 3163 | 22.7 | 37.8 | 6371 | 45.7 | 20.3 | 4398 | 31.6 | 23.5 |
| 0 |  |  |  |  |  |  |  |  |  |
| 1 | 1711 | 11.3 | 20.5 | 8957 | 59.4 | 28.6 | 4416 | 29.3 | 23.6 |
| 2+ | 1640 | 8.9 | 19.6 | 10940 | 59.2 | 34.9 | 5895 | 31.9 | 31.6 |
| unknown | 1850 | 17.0 | 22.1 | 5040 | 46.4 | 16.1 | 3972 | 36.6 | 21.3 |
| **Health insurance status**  **(primary and secondary payer)** | 111 | 19.0 | 1.3 | 306 | 52.5 | 1.0 | 166 | 28.5 | 0.9 |
| No insurance |  |  |  |  |  |  |  |  |  |
| Private only | 3147 | 16.8 | 37.6 | 9289 | 49.5 | 29.7 | 6329 | 33.7 | 33.9 |
| Medicare only or Medicare+Private | 3012 | 12.3 | 36.0 | 13620 | 55.8 | 43.5 | 7772 | 31.8 | 41.6 |
| Any Medicaid | 1807 | 16.3 | 21.6 | 6376 | 57.4 | 20.4 | 2922 | 26.3 | 15.6 |
| Any Military/Other Public | 166 | 6.9 | 2.0 | 1157 | 48.2 | 3.7 | 1077 | 44.9 | 5.8 |
| Unknown | 121 | 11.0 | 1.4 | 560 | 51.1 | 1.8 | 415 | 37.9 | 2.2 |
| **Patient seen at NCI-designated**  **cancer center for this tumor** | 6598 | 13.2 | 78.9 | 26864 | 53.7 | 85.8 | 16569 | 33.1 | 88.7 |
| not NCI |  |  |  |  |  |  |  |  |  |
| seen at NCI center | 1766 | 21.2 | 21.1 | 4444 | 53.4 | 14.2 | 2112 | 25.4 | 11.3 |
| **Marital status at diagnosis** | 1302 | 12.4 | 15.6 | 6027 | 57.3 | 19.3 | 3186 | 30.3 | 17.1 |
| never married |  |  |  |  |  |  |  |  |  |
| married/domestic partner | 4812 | 16.2 | 57.5 | 15307 | 51.4 | 48.9 | 9635 | 32.4 | 51.6 |
| separated, divorced, widow | 2019 | 12.5 | 24.1 | 9054 | 55.8 | 28.9 | 5139 | 31.7 | 27.5 |
| unknown | 231 | 12.3 | 2.8 | 920 | 49.1 | 2.9 | 721 | 38.5 | 3.9 |
| **Neighborhood Socioeconomic Status**  **(SES) quintile** |  |  |  |  |  |  |  |  |  |
| Q1 (lowest) | 1070 | 11.9 | 12.8 | 5066 | 56.4 | 16.2 | 2852 | 31.7 | 15.3 |
| Q2 | 1398 | 11.9 | 16.7 | 6582 | 56.2 | 21.0 | 3736 | 31.9 | 20.0 |
| Q3 | 1645 | 13.1 | 19.7 | 6958 | 55.3 | 22.2 | 3969 | 31.6 | 21.2 |
| Q4 | 1913 | 15.6 | 22.9 | 6518 | 53.1 | 20.8 | 3853 | 31.4 | 20.6 |
| Q5 (highest) | 2093 | 19.4 | 25.0 | 5132 | 47.5 | 16.4 | 3588 | 33.2 | 19.2 |
| unknown | 245 | 12.4 | 2.9 | 1052 | 53.1 | 3.4 | 683 | 34.5 | 3.7 |
